# A urokinase-associated outbreak of *Ralstonia mannitolilytica* bloodstream infections in haemodialysis patients in north-eastern Italy, January to April 2023

**DOI:** 10.2807/1560-7917.ES.2023.28.28.2300328

**Published:** 2023-07-13

**Authors:** Massimiliano Fabricci, Anaïs Trinca, Luca Talotti, Marina Busetti, Emmanouil Alexandros Fotakis, Christina Merakou, Raffaella Koncan, Annachiara Ghiotti, Camilla Negri, Vittorio Di Maso, Manuela Bosco, Alberto Antonelli, Marco Coppi, Gian Maria Rossolini, Claudia Giuliani, Enrico Scarpis, Barbara Gregoretti, Danilo Licastro, Roberto Luzzati, Venera Costantino, the multidisciplinary working group, Multidisciplinary working group, Fabio Barbone, Elisa Bedina, Tania Bottacin, Alice Cara, Leda Cipriani, Roberto Cocconi, Maryluz Cordova Luna, Pierlanfranco D’Agaro, Stefano Di Bella, Manuela Di Santolo, Stefano Dolce, Roberta Fais, Francesco Fontana, Antonietta Guerra, Claudia Lucarelli, Francesco Maraglino,, Massimiliano Martone, Cristina Maurel, Lucia Mian, Jacopo Monticelli, Alberto Pagotto, Ketty Parenzan, Patrizio Pezzotti, Alessandro Pipoli, Chiara Roni, Michela Sabbatucci,, Assunta Sartor, Paolo Schincariol, Carlo Tascini, Massimiliano Tosto, Gabriele Turello

**Affiliations:** 1 Medical Directorate, Trieste University Hospital (ASUGI), Trieste, Italy; 2 Clinical Department of Medical, Surgical and Health Sciences, University of Trieste, Trieste, Italy; 3 Microbiology Unit, Trieste University Hospital (ASUGI), Trieste, Italy; 4 ECDC Fellowship Programme, Field Epidemiology path (EPIET), European Centre for Disease Prevention and Control (ECDC), Stockholm, Sweden; 5 Italian National Institute of health (ISS), Rome, Italy; 6 ECDC fellowship Programme, Public Health Microbiology path (EUPHEM), European Centre for Disease Prevention and Control (ECDC), Stockholm, Sweden; 7 Department of Life Sciences, University of Trieste, Trieste, Italy; 8 Medical Directorate, Gorizia and Monfalcone Hospital (ASUGI), Gorizia and Monfalcone, Italy; 9 Nephrology and Dialysis Unit, Trieste University Hospital (ASUGI), Trieste, Italy; 10 Nephrology and Dialysis Unit, Gorizia and Monfalcone Hospital (ASUGI), Gorizia and Monfalcone, Italy; 11 Microbiology and Virology Unit, Careggi University Hospital, Florence, Italy; 12 Department of Experimental and Clinical Medicine, University of Florence, Florence, Italy; 13 Medical Directorate, Palmanova Hospital (ASUFC), Palmanova, Italy; 14 AREA Science Park, Trieste, Italy; 15 Infectious Diseases Unit, Trieste University Hospital (ASUGI), Trieste, Italy; 16 The members of this group are listed under Collaborators

**Keywords:** Outbreak, Ralstonia, mannitolilytica, urokinase, dialysis, bacteraemia, catheter, sepsis

## Abstract

An outbreak of *Ralstonia mannitolilytica* bloodstream infections occurred in four hospitals in north-eastern Italy, involving 20 haemodialysis patients with tunnelled central vascular catheter access. We identified as the outbreak source a batch of urokinase vials imported from India contaminated with *R. mannitolilytica.* Whole genome sequences of the clinical and urokinase strains were highly related, and only urokinase-treated patients were reported with *R. mannitolilytica* infections (attack rate = 34%; 95% confidence interval: 22.1–47.4). Discontinuation of the contaminated urokinase terminated the outbreak.

In January 2023, four patients undergoing haemodialysis at a tertiary care hospital in Italy were diagnosed positive for *Ralstonia mannitolilytica* bloodstream infections. By mid-April, a total of 20 case-patients were confirmed in four different hospitals in the Friuli-Venezia-Giulia Region, north-eastern Italy. The purpose of this rapid communication is to alert all healthcare providers about the presence of a contaminated batch of urokinase and to provide guidance regarding the management of *R. mannitolilytica* outbreaks. We present here the epidemiological and microbiological findings from the outbreak investigation. 

## Outbreak evolution

**Figure 1:**
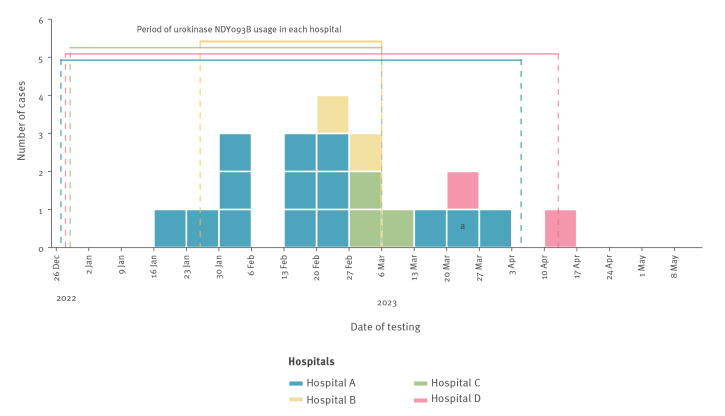
*Ralstonia mannitolilytica* bloodstream infections in haemodialysis patients in four hospitals, north-eastern Italy, January–April 2023 (n = 20)

^a^ Repeated positive blood culture of the index case.

In mid-January 2023, a patient receiving haemodialysis with an indwelling central vascular catheter (CVC) in hospital A developed fever and chills during their haemodialysis session. Following a positive blood culture for Gram-negative rods, *R. mannitolilytica* was identified as the causative pathogen. Within 14 days of the first case, four additional haemodialysis patients with CVC in hospital A presented fever during their sessions and tested positive for *R. mannitolilytica*. An outbreak was declared with a case defined as any haemodialysis patient with a CVC with at least one blood culture positive for *R. mannitolilytica,* regardless of symptom occurrence. Five weeks after the first case in hospital A, a symptomatic patient from hospital B also tested positive for *R. mannitolilytica*. Ten days later, a case was identified in hospital C and 3 weeks after that, a case was identified in hospital D. By 11 April 2023, systematic screening for *R. mannitolilytica* infection in patients with CVC had identified a total of 20 case-patients (16 symptomatic and four without symptoms or signs of infection), with one patient’s blood cultures testing positive repeatedly (Figure 1). On 7 April 2023, microbiological investigations identified *R. mannitolilytica* in a batch of urokinase manufactured in India, used in all the affected hospitals as a thrombolytic drug for the prevention and management of obstructed CVCs during different time periods between January and April 2023. Following the removal of the contaminated urokinase batch from clinical use, no additional *Ralstonia* spp. infections were identified in Friuli-Venezia-Giulia Region.

## Outbreak setting and case characteristics

All cases included in the outbreak investigation were reported by the dialysis units of four hospitals in the Friuli-Venezia-Giulia Region (north-eastern Italy, 1.2 million inhabitants). These hospitals receive materials and drugs from a single regional provider. During the outbreak (January–April 2023), a total of 332 patients (median age: 75 years; interquartile range: 66–82) underwent haemodialysis treatment in the affected hospitals with either a CVC or an AVF. The majority of cases (13/20) were identified in hospital A while the distribution of cases by sex and age group followed that of the overall patient population (Table 1). The attack rate among patients with a CVC was 11%. No cases were reported among patients with exclusive AVF treatment.

**Table 1:** Characteristics of cases with *Ralstonia mannitolilytica* bloodstream infection and overall haemodialysis patient population, four hospitals in north-eastern Italy, January–April 2023 (n = 332)

Characteristics	Cases (n = 20)	Overall (n = 332)
**Hospital**		
A	13	152
B	2	44
C	3	43
D	2	93
**Haemodialysis treatment **		
Patients with tunnelled CVC^a^	20	178
Patients who did not have a CVC^b^	0	154
**Sex **		
Females	6	105
Males	14	227
**Age group (years) **		
< 49	0	17
50–59	3	29
60–69	4	53
70–79	8	119
> 80	5	114

CVC: central vascular catheter.

^a^ Patients with a CVC at any time point between January and April 2023.

^b^ Patients who did not have a CVC at any time point during the study period were monitored for clinical symptoms but were not tested for *Ralstonia* infection.

## Microbiological investigations

Blood cultures obtained from both peripheral vein and CVC, and CVC tip cultures were performed with the BactAlert and BD Bactec automated systems. MALDI-TOF mass spectrometry identified *R. mannitolilytica* in all blood cultures from all patients with peripheral vein and CVC, including a patient co-infected with *R. mannitolilytica/Ralstonia pickettii*, and in 10 case-patient catheter tips. We also identified *R. pickettii* in two patients from the same hospital with CVC with no signs of infection (not included as cases here).

Environmental and pharmacological samples, including dialysis water, heparin, urokinase, saline solution, sodium citrate, chlorhexidine and epoetin alfa, were retrieved from the affected hospitals. Initially, all samples (including six urokinase vials from contaminated batch and two from a distinct batch) were inoculated on blood agar plates and on brain heart infusion (BHI) enrichment broth, incubated at 35 °C and 28 °C for 14 days. All samples collected until March 2023 tested negative for *Ralstonia* spp. From April 2023 onwards, BHI was substituted with blood culture aerobic bottles. Subsequently, *R. mannitolilytica* was identified in a batch of pristine monodose urokinase vials (batch no. NDY093B) administered to patients with CVC in all four hospitals, at different time points between January and April 2023 (Figure 1). We isolated *R. mannitolilytica* from three NDY093B batch samples collected from hospitals A, B and C, while two samples from hospital D tested negative. Saline solution used to dissolve lyophilised urokinase powder tested negative. All environmental and pharmacological samples were negative for *R. pickettii.*

**Figure 2:**
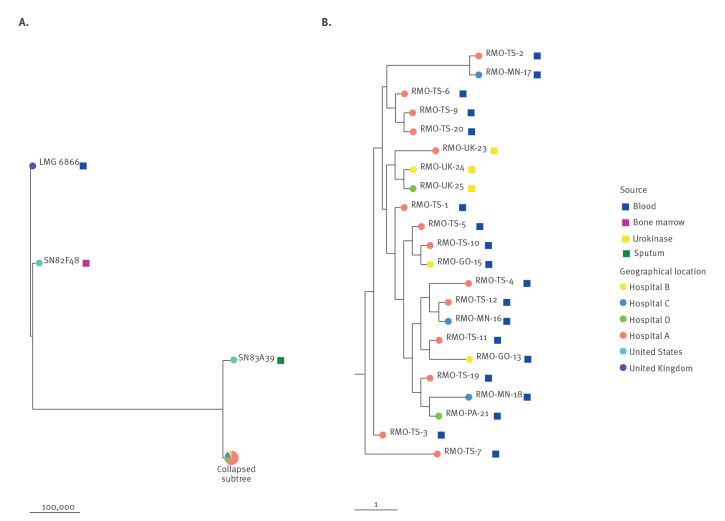
Phylogenetic analysis of *Ralstonia mannitolilytica* clinical isolates (n = 19) and urokinase vials (batch no. NDY093B; n = 3), four hospitals in north-eastern Italy, January–April 2023

Analysis by full-length 16S rRNA gene sequencing confirmed MALDI-TOF results for *R. mannitolilytica* and *R. pickettii* species identification. We performed whole genome sequencing (WHS) (Illumina platform [1]) on all *R. mannitolilytica* isolates from urokinase (n = 3) and 19 of the 20 case-patients (Bioproject ID PRJNA983316). The WGS species identification matched the above diagnostic analyses. Clonal relatedness was investigated by generation of core-genome single nucleotide polymorphisms (SNPs) using Snippy (v 4.6.0, https://github.com/tseemann/snippy) and the draft genome of RMO-TS-1 (the first *R. mannitolilytica* isolated from a patient in hospital A, National Center for Biotechnology Information (NCBI) accession no. SAMN35733911). After filtering elevated densities of base substitutions using Gubbins (v 3.2.1), the resulting variable positions were used to infer maximum likelihood trees with IQ-TREE (v 2.2.0.3) using a general time reversible model. The genomes of strains SN82F48 (the NCBI reference genome for *R. mannitolilytica*, accession no. GCF_000954135.1), LMG 6866 (the *R. mannitolilytica* type strain, NCBI accession no. ERR5548255) and SN83A39 (RefSeq GCF_001628775.1) were also included as outliers representative of the same species. Comparative genomic analysis confirmed that the case-patient *R. mannitolilytica* isolates were closely related to each other (0–4 SNPs) and with those isolated from the three urokinase vials (0–3 SNPs), while being distantly related to the *R. mannitolilytica* reference strains included in the analysis as representatives of the same species (21,570–128,663 SNPs), underscoring the notable genomic diversity existing within this species (Figure 2).

Branch lengths are reported in substitutions (point mutations) per genome. Genomes of strains LMG 6866 (*R. mannitolilytica* type strain) and SN82F48 (complete NCBI reference genome for *R. mannitolilytica*) and SN83A39 (RefSeq GCF_001628775.1) were included in the analysis as references representatives of the same species. Panel B shows the details of the collapsed subtree including the outbreak isolates.

## Attack rates in haemodialysis patients with central vascular catheter

During the outbreak, the contaminated urokinase (i.e. batch *NDY093B)* was used in 59 patients (from a total of 178 patients with CVC) from all hospitals, partially overlapping with the use of non-contaminated batches. Attack rates of all patients exposed to urokinase are described in Table 2. 

**Table 2:** Urokinase exposure attack rates, *Ralstonia mannitolilytica* bloodstream infection outbreak in haemodialysis patients with CVC, four hospitals in north-eastern Italy, January–April 2023 (n = 178)

Hospital	Exposed to urokinase^a^		
	Cases	Total	AR (95% CI)
A	13	33	39% (22.9–57–4)
B	2	7	29% (3.7–71.0)
C	3	8	38% (8.5–75.6)
D	2	11	18% (2.3–51.8)
**Overall**	**20**	**59**	**34% (22.1–47.4)**

CI: confidence interval; AR: attack rate.

^a^ Urokinase exposure corresponds to ≥ 1 exposure events to contaminated or non-contaminated urokinase solution.

95% CI were calculated using exact methods.

None of the 119 patients (55 patients from hospital A, 15 from hospital B, 19 from hospital C and 30 from hospital D) with CVC who were not exposed to urokinase tested positive for *R. mannitolilytica*. Patients who did not have a CVC at any time point during the study period were not exposed to urokinase. Increased exposure to urokinase (i.e. ≥ 11 urokinase treatment sessions compared with < 10) was associated with increased risk of *R. mannitolilytica* infection (17/29; 58.6% vs 3/30; 10%, risk ratio (RR) = 5.9; 95% CI: 1.9–17.9; p = 0.01).

## Antimicrobial susceptibility in human samples

Antimicrobial susceptibility testing was performed on all isolates by microdilution (Sensititre, Thermofisher). All isolates tested resistant to meropenem (minimum inhibitory concentration (MIC) ≥ 16 mg/L), piperacillin/tazobactam (MIC ≥ 32), ceftazidime (MIC = 32), amikacin (MIC > 16), colistin (MIC > 8), aztreonam (MIC > 32) and ceftolozane/tazobactam (MIC > 8). The MICs for cefepime and imipenem ranged from 4 to 16. Fluoroquinolones (ciprofloxacin and levofloxacin) had low MICs (≤ 0.12) for all isolates.

## Case treatment, local control measures and outcomes

All patients with a positive blood culture for *R. mannitolilytica* received antibiotic therapy based on the antibiotic susceptibility results, including ciprofloxacin as outpatient regimen and cefepime plus ciprofloxacin as inpatient regime. In addition, 18 of the 20 cases underwent prompt CVC removal. Sixteen cases had a full recovery, while *R. mannitolilytica*-related sepsis was identified as the probable cause of death in one case. Three cases died due to unrelated causes, soon after resolution of the *R. mannitolilytica* infection. Upon the detection of urokinase samples positive for *R. mannitolilytica,* use of the monodose urokinase vials was discontinued, and the corresponding urokinase products were replaced with other drugs. The outbreak ended with the implementation of these changes (Figure 1).

## National and international communication of the outbreak

The medical director of hospital A informed initially (16 February 2023) the Friuli-Venezia-Giulia regional authorities of the ongoing outbreak and subsequently asked for support from the Italian National Institute of Health (ISS). The cases were notified (27 April 2023) to the Italian Ministry of Health (MoH) who reported this immediately to all the Italian regional focal points both for health prevention and antimicrobial resistance by an informative note, as well as informing other countries through the Early Warning and Response System of the European Union (EWRS) when the source of infection was identified. At that time, the MoH checked that additional cases of hospital-associated *Ralstonia* spp. infections were reported neither by other regions in Italy, nor through EWRS in other countries in the European Union and European Economic Area (EU/EEA), and informed Friuli-Venezia-Giulia Region and ISS accordingly. After the contaminated urokinase batch was identified, the Friuli-Venezia-Giulia Region alerted the Italian Medicines Agency (AIFA) of the positivity of this urokinase batch and the size and severity of the ongoing outbreak. Subsequently, AIFA informed all hospital pharmacies in Italy, and the World Health Organization, which as a matter of practice is responsible for advising the supplier. 

## Discussion 

*Ralstonia mannitolilytica* is a non-fermenting aerobic Gram-negative rod belonging to the bacterial genus *Ralstonia* found in humid environments such as water, soil and plants [2-4]. Four Ralstonia species are recognised as opportunistic human pathogens, including *R. pickettii*, *R. mannitolilytica*, *R. insidiosa* and *R. solanacearum*, with the first being the most clinically prevalent [3]. Nonetheless, during the past 10 years, several hospital outbreaks have been attributed to *R. mannitolilytica* infection worldwide [4-9], two of which occurred in dialysis units [7,9].

We identified 20 cases of *R. mannitolilytica* bloodstream infection during this multi-hospital outbreak, while no *R. mannitolilytica* isolates had previously been detected in any of the affected hospitals. Microbiological investigations identified the outbreak source in a specific batch of pristine monodose urokinase vials (NDY093B), purchased from an Indian company in December 2022, before the outbreak. This company served as an ad hoc urokinase supplier to the Friuli-Venezia-Giulia Region hospitals due to a concurrent urokinase supply shortage in Europe. It is likely that urokinase contamination occurred during the manufacturing phase as *R. mannitolilytica* was detected in three monodose vials and existing literature indicates that *Ralstonia* spp. can pass through the 0.2 μm filters regularly used for product sterilisation [4].

While in previous nosocomial *Ralstonia* spp. outbreaks, predominantly water but also disinfectants, antiseptics, saline solution and heparin syringes/bottles have been hypothesised or incriminated as the outbreak source [6,10-12], this is the first time *Ralstonia* bacteria have been detected in urokinase vials. Importantly, the source of the outbreak was only identified following a change in the sample culturing protocol, illustrating that enriched blood culture aerobic bottles plus blood agar are the best approach for bacterial isolation in this type of drug. Although the source of the *R. mannitolilytica* outbreak was successfully identified, there is no clear explanation for the concurrent circulation of *R. pickettii* in a limited number of patients.

Sixteen of 20 cases in this outbreak were symptomatic, presenting chills and/or fever during dialysis or at a later time point. Immediate clinical symptom onset upon exposure to contaminated urokinase or lagged symptom onset attributed to CVC colonisation by *R. mannitolilytica* and detachment-dissemination of the infectious agent during subsequent dialysis procedures are both plausible and in line with the known increased risk of CVC-related infections compared with AVF [4,13,14]. 

Following the introduction of the contaminated urokinase in the affected hospitals, most patients with CVC receiving urokinase treatment (n = 59) were exposed to the contaminated batch. However, non-contaminated urokinase batches were also used until the end of February. As a limitation of our study, we recognise that the calculated RR may slightly underestimate the true RR attributed to contaminated urokinase exposure, due to an overestimation of the true number of exposed patients.

## Conclusion 

Our findings suggest that in contexts of *R. mannitolilytica* case clusters in haemodialysis units, prime outbreak source suspects requiring investigation should include urokinase solutions used for the management of patients with CVCs. This also applies to other hospital procedure settings using urokinase, including intravenous treatment of pulmonary embolism and myocardial infarction. Case notification to the MoH allowed for prompt information sharing between local and central health authorities and for nationwide and EU/EEA-wide alerts. Risk assessments are ongoing in other regions of Italy to evaluate patient exposure to the contaminated urokinase batch, in order to initiate *R. mannitolilytica* infection screening schemes and complementary control measures where necessary. The European Centre for Disease prevention and Control encourages EU/EEA countries to investigate whether urokinase produced by the implicated supplier has been purchased and/or used and if cases have occurred in their country [15]. 
